# Resveratrol mediates mechanical allodynia through modulating inflammatory response via the TREM2-autophagy axis in SNI rat model

**DOI:** 10.1186/s12974-020-01991-2

**Published:** 2020-10-21

**Authors:** Yaping Wang, Yu Shi, Yongquan Huang, Wei Liu, Guiyuan Cai, Shimin Huang, Yanyan Zeng, Siqiang Ren, Hongrui Zhan, Wen Wu

**Affiliations:** 1 Department of Rehabilitation, Zhujiang Hospital, Southern Medical University, Guangzhou, 510282 Guangdong China; 2 Guangdong-Hong Kong-Macao Greater Bay Area Center for Brain Science and Brain-Inspired Intelligence; Key Laboratory of Mental Health of the Ministry of Education; Guangdong Province Key Laboratory of Psychiatric Disorders, Southern Medical University, Guangzhou, 510515 Guangdong China; 3 Department of Ultrasound, The Fifth Affiliated Hospital of Sun Yat-sen University, Zhuhai, 519000 Guangdong China; 4 Department of Rehabilitation, Shenzhen University General Hospital, Shenzhen, 518005 Guangdong China; 5 Department of Rehabilitation, The Fifth Affiliated Hospital of Sun Yat-sen University, Zhuhai, 519000 Guangdong China

**Keywords:** TREM2, Resveratrol, Microglia, Autophagy, Neuropathic pain

## Abstract

**Background:**

Neuropathic pain (NeuP) is a chronic and challenging clinical problem, with little effective treatment. Resveratrol has shown neuroprotection by inhibiting inflammatory response in NeuP. Recently, the triggering receptor expressed on myeloid cells 2 (TREM2) expressed by microglia was identified as a critical factor of inflammation in nervous system diseases. In this study, we explored whether resveratrol could ameliorate neuroinflammation and produce anti-mechanical allodynia effects via regulating TREM2 in spared nerve injury rats, as well as investigated the underlying mechanisms.

**Methods:**

A spared nerve injury (SNI) rat model was performed to investigate whether resveratrol could exert anti-mechanical allodynia effects via inhibiting neuroinflammation. To evaluate the role of TREM2 in anti-neuroinflammatory function of resveratrol, lentivirus coding TREM2 was intrathecally injected into SNI rats to activate TREM2, and the pain behavior was detected by the von Frey test. Furthermore, 3-methyladenine (3-MA, an autophagy inhibitor) was applied to study the molecular mechanisms of resveratrol-mediated anti-neuroinflammation using Western blot, qPCR, and immunofluorescence.

**Results:**

The TREM2 expression and number of the microglial cells were significantly increased in the ipsilateral spinal dorsal horn after SNI. We found that intrathecal administration of resveratrol (300ug/day) alleviated mechanical allodynia; obviously enhanced autophagy; and markedly reduced the levels of interleukin-1β, interleukin-6, and tumor necrosis factor-α in the ipsilateral spinal dorsal horn after SNI. Moreover, the number of Iba-1^+^ microglial cells and TREM2 expression were downregulated after resveratrol treatment. Intrathecal administration of lentivirus coding TREM2 and/or 3-MA in those rats induced deficiencies in resveratrol-mediated anti-inflammation, leading to mechanical allodynia that could be rescued via administration of Res. Furthermore, 3-MA treatment contributed to TREM2-mediated mechanical allodynia.

**Conclusions:**

Taken together, these data reveal that resveratrol relieves neuropathic pain through suppressing microglia-mediated neuroinflammation via regulating the TREM2-autophagy axis in SNI rats.

## Background

Neuropathic pain (NeuP) is a chronic and complex pain caused by nerve injury, usually due to cancerous, chemical, infectious, or traumatic injury. NeuP, affecting 7-10% of the general population, is manifested as allodynia, hyperalgesia, and spontaneous pain [1, 2]. Neuroinflammation has been described as the primary etiology of NeuP, contributing to peripheral and central hypersensitization [3]. Recently, increasing studies investigating NeuP have described the vital role of microglia in the spinal cord [4]. Microglia, as natural immune cells in the central nervous system (CNS), are necessary to maintain the support, protection, and surveillance [5]. Mounting studies have indicated that dramatic microgliosis and microglia activation in the spinal dorsal horn (SDH) aggravates neuroinflammation and mechanical hypersensitivity by releasing various pro-inflammatory cytokines, including interleukin-1β (IL-1β), interleukin-6 (IL-6), and tumor necrosis factor-α (TNF-α). In contrast, suppression of microglia activation improved mechanical hypersensitivity [6, 7]. However, the specific mechanism of microglia-induced neuroinflammation has not been fully addressed in previous studies and the therapy for NeuP remains an unsolved problem.

Resveratrol, extracted from herbal, is a natural polyphenol with beneficial effects, including anti-aging, anti-cancer, anti-inflammation, and anti-oxidative effects [8]. Evidences reveal that resveratrol exerts neuroprotective effects through inhibiting neuroinflammation in neurological diseases, such as Alzheimer’s disease, ischemic stroke, and traumatic brain injury [9–11]. Recent studies have shown that under the NeuP condition, resveratrol inhibited the release of pro-inflammatory cytokines, such as IL-1β, IL-6, TNF-α [12]. More recent studies demonstrated that resveratrol reduced inflammatory damage by suppressing microglia activation [13, 14]. Interestingly, resveratrol ameliorated microglia-mediated inflammation by promoting autophagy in inflammation diseases, such as gout, bone cancer, atherosclerosis, and neuropathic pain [15–17]. However, in dissecting these effects, we do not yet have a clear understanding of the effects of resveratrol on NeuP and the extent to which they depend on the neuroinflammatory mechanism.

Macroautophagy (herein, autophagy) is known to remove damaged organelles and aggregated proteins to sustain cell survival, immunity, and inflammation [18]. Roles for autophagy has been investigated in inflammatory disease, cancers, and neurodegenerative disorder such as Alzheimer’s disease and Parkinson’s disease. Autophagy has been linked to many aspects of innate immune signaling, antigen presentation, and lymphocyte [19]. A large body of previous works has suggested that autophagy is highly associated with the progression of NeuP, which is explored as a protective mechanism [20]. Moreover, autophagy has been associated with microglia-induced inflammation in NeuP [21, 22]. In addition, there is evidence that TREM2 is involved in autophagic flux in microglia [5].

The triggering receptor expressed on myeloid cells 2 (TREM2), a cell-surface receptor mainly expressed on microglia in the CNS, has been implicated in various diseases associated with Alzheimer’s disease, Nasu-Hakola disease, and neuropathic pain. The interaction of TREM2 and DNAX-activating protein of 12 kDa (DAP12), a TREM2-associated transmembrane adaptor, has been implicated in intracellular signal transduction, mediating immune response, cellular metabolism, and proliferation [23]. TREM2/DAP12 exacerbated neuropathic pain by increasing pro-inflammatory factor secretion from microglia [24]. Given the critical role of TREM2 in microglia in neuropathic pain, we hypothesized that resveratrol might alleviate neuroinflammation-triggered mechanical allodynia via TREM2-mediated autophagy.

In the present research, we revealed that the TREM2-autophagy pathway was activated, which induced the microglia-mediated inflammatory response in the spinal cord of spared nerve injury (SNI) rats. In addition, administration of resveratrol attenuated neuroinflammation and improved mechanical allodynia in SNI rats through inhibiting autophagy via downregulating TREM2.

## Materials and methods

### Animals

Adult male Sprague-Dawley rats (200-250 g) were obtained from the Southern Medical University Animal Center (Guangzhou, China). They were housed at a standard temperature of 24 ± 1 °C under a 12 h light-dark cycle (darks on from 7:00 pm to 7:00 am) with free access to food and water. All experiments were conducted in accordance with the National Institutes of Health Guide for the Care and Use of Laboratory animals in rigorous line with International Association for the Study of Pain guidelines. Our research was approved by the Southern Medical University Animal Ethics Committee. The behavioral tests were performed by experimenters who were blinded to the experimental group.

### The neuropathic pain model

Rats were anesthetized by intraperitoneal injection with 1% pentobarbital. The spared nerve injury model was previously described in rats [25]. Briefly, a small incision was made at the lateral skin of the thigh to expose the sciatic nerve and its three branches: the sural, common peroneal, and tibial nerves. The tibial and common peroneal nerves were carefully ligated and transected and the intact sural nerve was preserved. When these nerves were ligated, rapid contraction and tremor of hind limbs were shown in the ipsilateral side, which suggested successful establishment of the SNI model. The sham-operated group was performed as above without ligation or transection. The wound was closed, with 6.0 silk sutures to close the muscles and 5.0 silk sutures to close the skin.

### Surgery and drugs administration

Rats were deeply anesthetized by intraperitoneal (i.p.) injection of 1% pentobarbital sodium. After the L5 spinous was removed, the PE-10 catheter filled with sterile saline was then inserted into the intervertebral space between the lumbar 5 (L5) and L6 level of the spinal cord. The correct catheter location was described as the promptly bilateral paralysis of hindlimbs caused by the administration of 2% lidocaine, and this effect disappeared within 30 min. Rats with abnormal behavior, such as paralysis and infection, were excluded. The tail-flick response was used as an indicator of the successful injection. The animals were allowed to recover for at least 4 days.

SNI rats were randomly divided into six groups: SNI, SNI + NS, SNI + Res, SNI + Res + Lv-TREM2, SNI + Res + 3-MA, SNI + Res + Lv-TREM2 + 3-MA groups (*n* = 6/group). Resveratrol (Res) and 3-methyladenine (3-MA) were purchased from Sigma-Aldrich (USA). The lentiviral vector coding TREM2 (Lv-TREM2) and control vector were provided form Guangzhou FulenGen Co., Ltd. (Guangzhou, China). The SNI group was only inserted into the catheter. According to a previous study [26], the SNI + Res group was intrathecally (i.t.) injected with resveratrol (300 ug/day, 2 consecutive days from 6 to 7 d after SNI), whereas the SNI + NS group was i.t. injected with volume-matched normal saline (NS). To investigate the function of TREM2 or autophagy, the 10 ul Lv-TREM2 (1 × 10^9^ transduction unit, TU/ml) was i.t. injected into rats for 10 days before SNI surgery, and 10ul 3-MA (autophagy inhibitor, Selleck, 50uM) were i.t. injected into rats on postoperative day 6 and 7.

### Von Frey test

As previously described [27], mechanical sensitivity was assessed using von Frey test hairs (0.6, 1.0, 1.4, 2.0, 4.0, 6.0, 8.0, and 10.0 g) (Stoelting, Wood Dale, Illinois, USA). All the behavioral tests were performed blindly. Briefly, before the test, each rat was placed into a transparent chamber for 30 min adaption followed by placed on a metal mesh floor. A von Frey hair was then applied to stimulate their hind paws and held for approximately 3–4 s with a 10 min interval between tests. A test began with the application of 4.0 g von Frey hair. Quick withdrawal of the hind paw upon stimulation was considered as a positive response. Final results were converted to a 50% withdrawal threshold (PWT) using the Dixon’s up-and-down method [28].

### qRT-PCR

Total RNA from the spinal dorsal horn of the ipsilateral or contralateral L4–L6 segments washed by RNase-Free water was isolated using Trizol (Invitrogen, USA) according to the manufacturer’s instruction. The RNA was then quantified using a Nanodrop spectrophotometer (Thermo Fisher Scientific, USA). The cDNA was synthesized using a PrimeScript^TM^ RT reagent Kit with gRNA (Takara, Japan). Gene level was evaluated using the SYBR green PCR master mix (Thermo Fisher Scientific, USA) in an ABI 7500 Fast qPCR system (Applied Biosystems, USA) according to manufacture instruction. Primers of targeting genes were synthesized (TREM2 (sense primer: 5′-GGA ACC GTC ACC ATC ACT CT-3′, antisense primer: 5′-ATG CTG GCT GCA AGA AAC TT-3′); TNF-α (sense primer: 5′-GTG GAA CTG GCA GAA GAG GC-3′, antisense primer: 5′-AGA CAG AAG AGC GTG GTG GC-3′); IL-1β (sense primer: 5′-CTG TGT CTT TCC CGT GGA CC-3′, antisense primer: 5′-CAG CTC ATA TGG GTC CGA CA-3′); IL-6 (sense primer: 5′-TTC CAT CCA GTT GCC TTC TT-3′, antisense primer: 5′-CAG AAT TGC CAT TGC ACA AC-3′); GAPDH (sense primer: 5′-AGT GTT TCC TCG TCC CGT AGA-3′, antisense primer: 5′-TTG CCG TGA GTG GAG TCA TAC-3′)). The GAPDH, a housekeeping gene, was used as control. Results were normalized to GAPDH and examined using the comparative CT method.

### Western blot

Rats were sacrificed with an intraperitoneal injection of sodium pentobarbital. The spinal dorsal horn of the L4–L6 segments were promptly removed and put into the ice-cold radio immunoprecipitation assay (RIPA) lysis buffer (including protease inhibitor cocktail), and then the spinal dorsal horn was divided into two part: ipsilateral and contralateral section. The supernatants were obtained after centrifuged with 14,000×*g* for 25 min at 4 °C. The total protein concentration was measured using the bicinchoninic acid (BCA) Protein Assay Kit (Thermo-Fisher, Rockford, IL, USA). Samples were then heated to 98 °C for 15 min. Equal amounts of total protein were loaded into lanes and separated on 12% SDS-PAGE gel. Then the proteins were transferred onto 0.45 μm polyvinylidene fluoride (PVDF) membranes. The membrane was blocked with 5% non-fat milk in tris-buffered-saline with tween (TBST) for 1.5 h at room temperature followed by incubated with the specific primary antibody for 12-14 h at 4 °C. The antibodies were anti-TRME2 (1:2000, R&D Systems, MAB17291); anti-P62 (1:4000, Cell Signaling Technology, #8025); anti-LC3 (1:5000, Cell Signaling Technology, #4108); anti-IL-1β (1:1000, Santa Cruz, sc-52012); anti-TNF-α (1:1000, Santa Cruz, sc-52746), anti-IL-6 (1:1000, Cell Signaling Technology, #12153), and anti-β-actin (1:1000, Cell Signaling Technology, #4970). Then, the blots were incubated with the corresponding secondary antibody (Cell Signaling Technology) for 1.5 h at room temperature. The blots were probed with Plus-ECL reagents (PerkinElmer, USA) and visualized with the image system (Bio-Rad). All of the results were quantified using the software ImageJ.

### Immunofluorescence

Rats were deeply anesthetized with 1% pentobarbital sodium by i.p. injection and quickly perfused with 0.9% saline followed by 4% formaldehyde. The spinal cords of the L4-L6 section were extracted and fixed in 4% formaldehyde for 12 h at 4 °C. Samples were immersed in 30% sucrose in 0.1 M PBS at 4 °C for 3-4 days. Then, the 25-um-thick sections of spinal samples were prepared using a cryostat (CM1950; Leica, Germany). After blocking in 3% bovine serum albumin (BSA) solution containing 1% Triton X-100 for 1.5 h at room temperature, sections were washed in PBS and then stained with primary antibodies (anti-Iba-1 (1:500, Abcam, ab15690), anti-TREM2 (1:1000, R&D System, MAB17291)) for 12-14 h at 4 °C followed by corresponding secondary antibodies (1:1000, Invitrogen). Images were captured using a confocal microscope (Nikon, Japan). Immunofluorescence density was analyzed by using the software ImageJ.

### Statistical analysis

All of the results were shown as the means ± standard error of mean (SEM). Our results were analyzed using the GraphPad Prism 7.0 software. Potential differences between any given two groups were evaluated using independent-sample *t* tests. One-way analysis of variance (ANOVA) followed by Tukey’s post hoc test was used to compare differences between any given two groups. Comparisons of multiple groups were performed by two-way repeated-measures ANOVA followed by Tukey’s post hoc test. The significance threshold for all of the experiments was set at *P* < 0.05.

## Results

### TREM2 is upregulated in microglia in the ipsilateral spinal dorsal horn of SNI rats

**Fig. 1:**
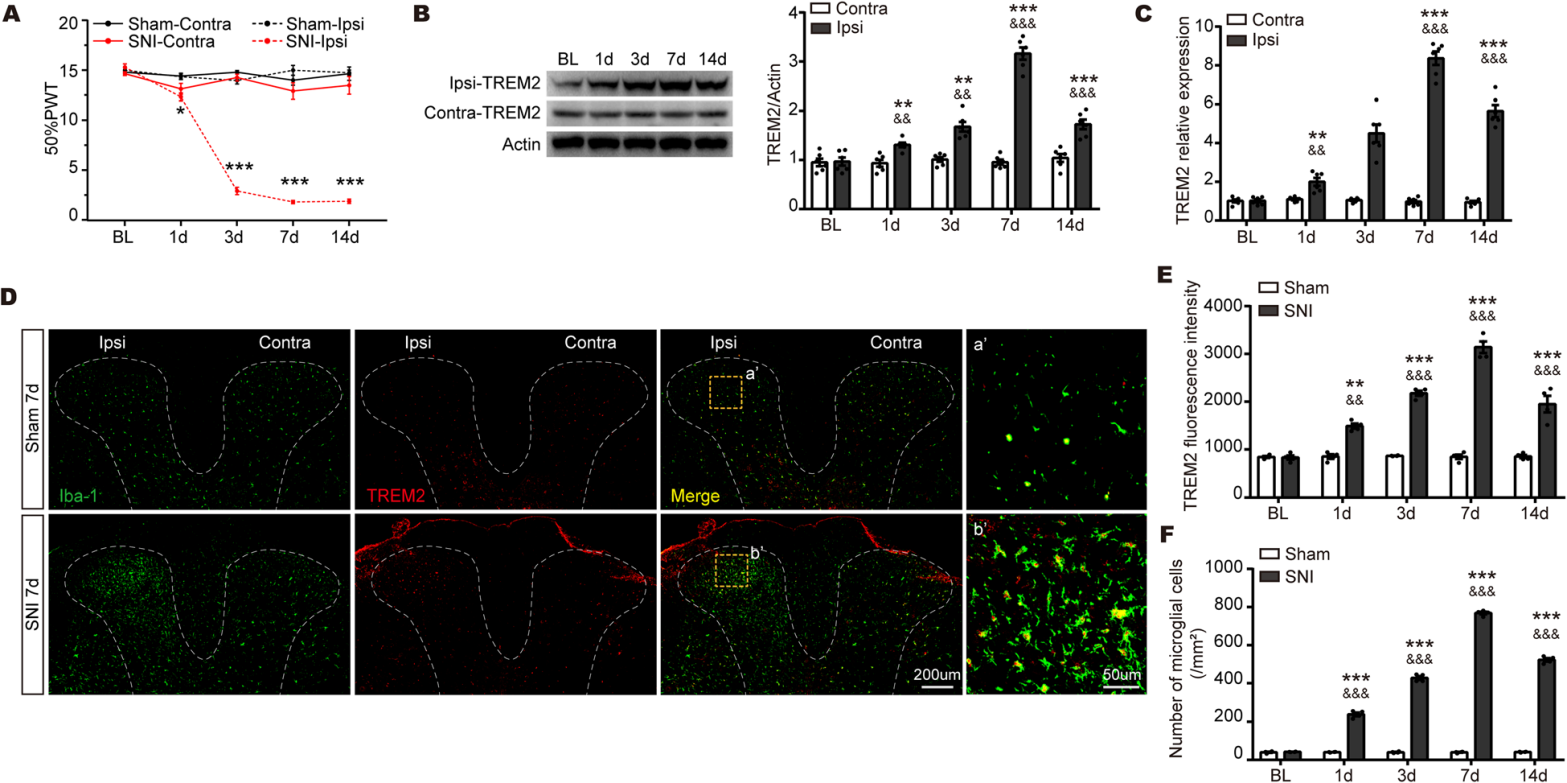
The expression of TREM2 in microglia is upregulated in the ipsilateral SDH after SNI surgery. **a** The 50% PWT in the ipsilateral (Ipsi) and/or contralateral (Contra) hind paw was evaluated by von Frey test before and after SNI in Sham or SNI rats. Data were presented as the mean ± SEM. *p < 0.05, ***p < 0.001 vs. SNI-Contra group. **b** Representative blots and expression of TREM2 was analyzed by Western blot in the ipsilateral (Ipsi) and/or contralateral (Contra) SDH at post-operative day 0-14 following spared nerve injury (SNI) (*n* = 6/group). **c** The expression of TREM2 mRNA was detected by qPCR in the SDH at different time points following SNI. Results were normalized to the housekeeping gene Gapdh (*n* = 6/group). **p < 0.01, ***p < 0.001 vs. Ipsi, &&*p* < 0.01, &&&*p* < 0.001 vs Contra. **d** Representative confocal images of double immunostaining showing colocalization of Iba-1 (green) and TREM2 (red) signals in the SDH at 7 days in Sham and SNI rats. The yellow dotted boxes showed areas of higher magnification in the ipsilateral SDH (a’ and b’). Original magnification, ×20. Scale bar, 200 μm (**d**), 50 μm (a’ and b’; **d**). **e**, **f** Quantification of TREM2 fluorescence intensity (**e**) and Iba1-positive microglial numbers (**f**) in the ipsilateral SDH at all time points in Sham and SNI rats (*n* = 4 per group). All results were presented as the mean ± SEM. **p < 0.01, ***p < 0.001 vs. SNI. &&*p* < 0.01, &&&*p* < 0.001 vs Sham

To evaluate the effects of the spared nerve injury (SNI) rat model of neuropathic pain, the von Frey test was performed before and after SNI surgery. Compared with the contralateral hind paw of SNI rats, the 50% paw withdrawal threshold (PWT) was decreased in the ipsilateral hind paw from day 1 to day 14 in SNI rats and reached a peak on day 7 (Fig. 1a). A significant difference was never observed in the ipsilateral and contralateral of Sham rats. These results suggest that the rat model of NeuP was successfully established.

To analyze the role of TREM2 in neuropathic pain, the spared nerve injury (SNI) model was applied to stimulate neuropathic pain caused by peripheral nerve injury. The expression of TREM2 in the spinal dorsal horn (SDH) were detected before and after SNI surgery. As shown in Fig. 1b, TREM2 protein was markedly upregulated and reached a peak in the ipsilateral SDH at day 7 after SNI, whereas no significant difference was observed in the contralateral SDH. We then evaluated TREM2 mRNA using qPCR. In line with the protein expression of TREM2, the level of TREM2 mRNA showed a similar pattern (Fig. 1c). To evaluate cell types that express TREM2 in the SDH of SNI rats, double-labeling immunofluorescence staining was performed. TREM2 was mainly colocalized with Iba-1-positive microglia in the ipsilateral of SDH (Fig. 1d), rarely in neuron, not with GFAP (astroglia marker) (Fig. S1). Consistent with Western blot results, TREM2 was significantly activated after SNI surgery (Fig. 1e). A significant increase in the number of microglia was detected in the ipsilateral SDH in SNI rats compared to that of the contralateral SDH (Fig. 1f). In summary, these results show that TREM2 is mainly expressed in microglia and activated in the SDH after SNI.

### Autophagy is obstructed in the ipsilateral spinal dorsal horn after SNI

**Fig. 2:**
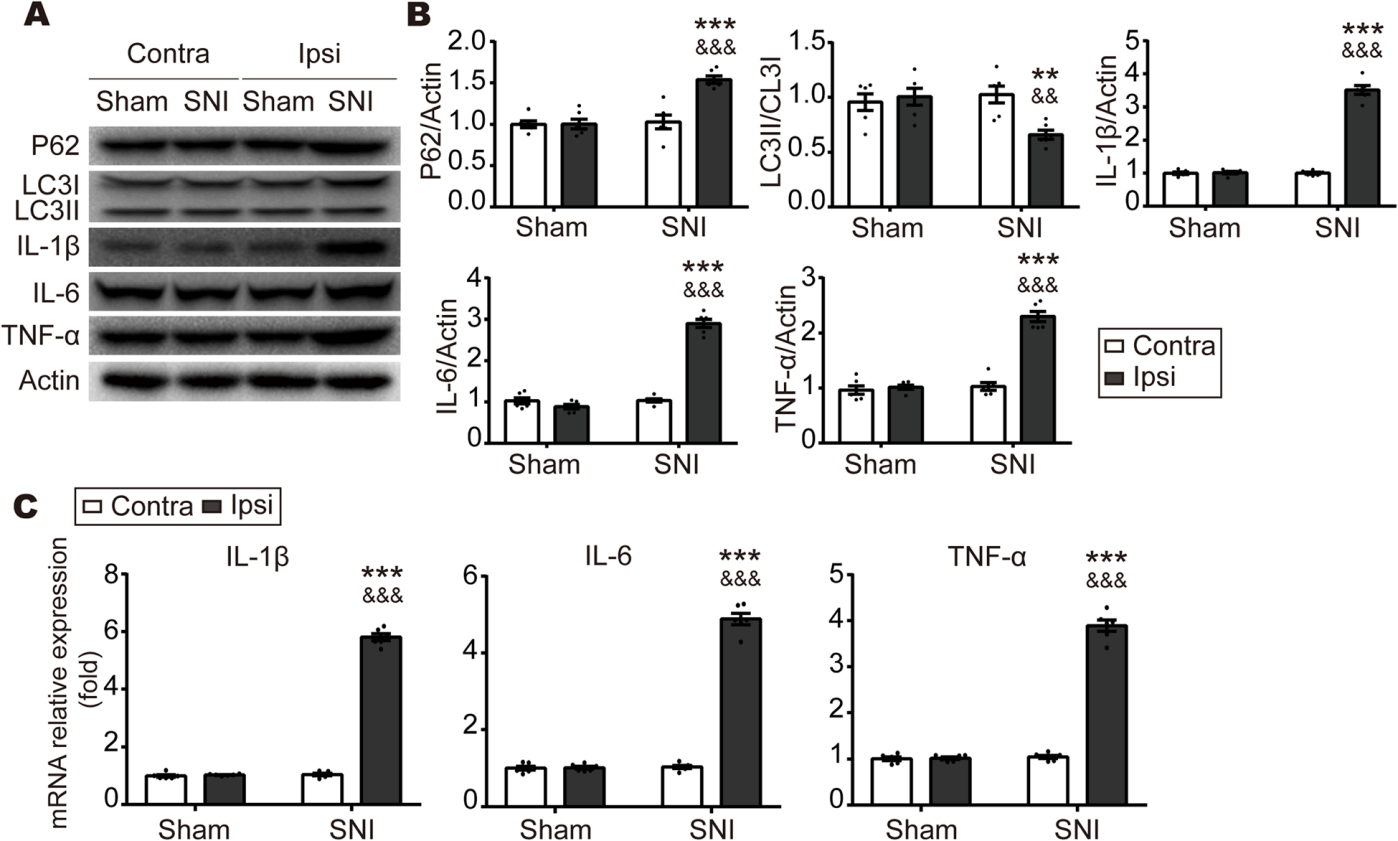
Autophagy is inhibited in the ipsilateral SDH following SNI. **a**, **b** Representative blots and quantification of P62, LC3II/LC3I, IL-1β, IL-6, and TNF-α in the contralateral or ipsilateral SDH at 7 days in Sham and SNI rats. **c** qPCR analysis of IL-1β, IL-6, and TNF-α levels in the contralateral or ipsilateral SDH at 7 days in Sham and SNI rats. Data were normalized to GAPDH. *n* = 6 rats per group. Values were mean ± SEM. ***p < 0.001 vs. SNI-Contra group. &&&*p* < 0.001 vs Sham-Ipsi group

To investigate the involvement of autophagy during neuropathic pain, we examined the expression of autophagy-associated markers, P62 and LC3, by Western blot in the ipsilateral SDH in SNI rats at 7 days. As shown in Fig. 2a, b, the expression of P62 was significantly increased, whereas the ratio of lipidated LC3II to non-lipidated LC3I was markedly decreased in the ipsilateral SDH in SNI rats compared to the contralateral spinal cord. No significant change was observed in Sham rats. Then, we also examined the expression of pro-inflammatory cytokines by Western blot and qPCR. The protein and mRNA levels of TNF-α, IL-1β, and IL-6 were dramatically augmented in the ipsilateral SDH of SNI rats, compared to the contralateral SDH of SNI rats or the ipsilateral SDH of Sham rats (Fig. 2a-c). Collectively, these data suggest that autophagy is inhibited during the pathology of inflammation in neuropathic pain.

### Resveratrol alleviates mechanical allodynia in SNI rats

**Fig. 3:**
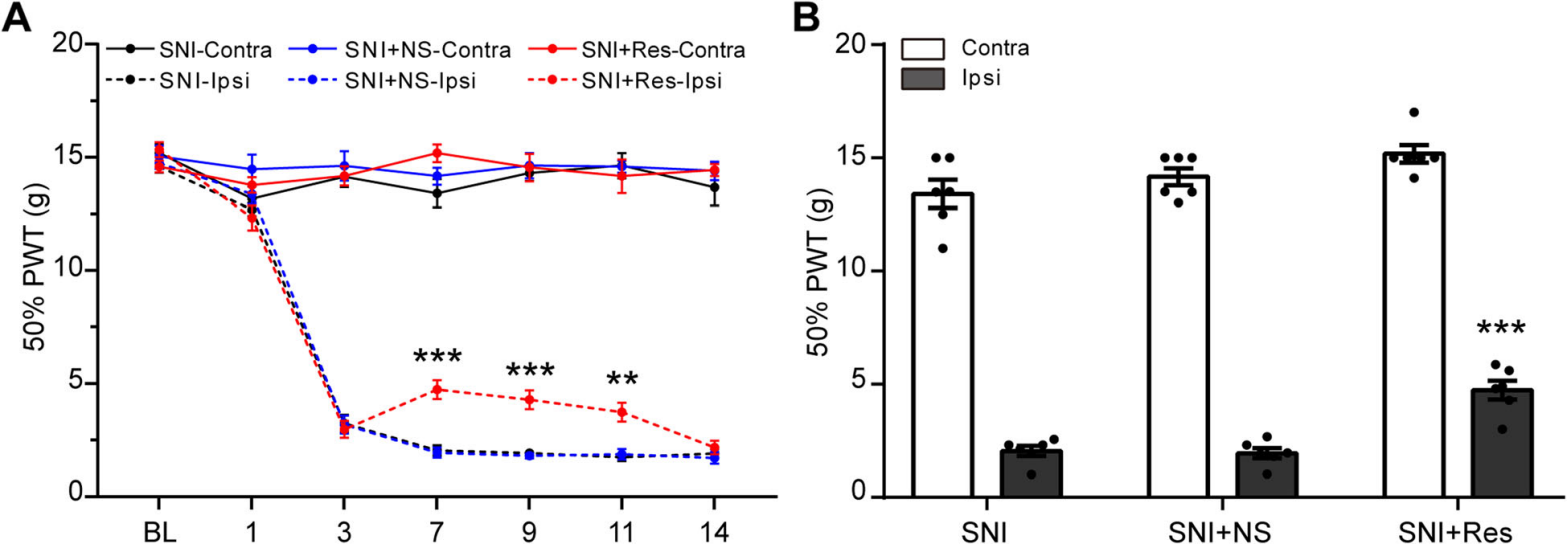
Resveratrol attenuates mechanical allodynia after SNI surgery. **a** The 50% PWT in the ipsilateral or contralateral side of SNI rats at all time points after intrathecal administration of resveratrol (Res) or normal saline (NS). **b** The 50% PWT in the ipsilateral side on day 7 from the three groups shown in (**a**). Data were presented as mean ± SEM. *n* = 6 rats per group. **p < 0.01 vs SNI + NS Ipsi group

To determine the effect of resveratrol (Res) on neuropathic pain characterized by pain hypersensitivity, the mechanical allodynia was evaluated in SNI rats at 0, 1, 3, 7, 9, 11, and 14 days using 50% paw withdrawal threshold (PWT) by the von Frey test after resveratrol treatment. As illustrated in Fig. 3a and b, a significant increase in the ipsilateral side was observed at 7 days in SNI + Res group, compared with SNI + NS group (Fig. 3a). There was no prominent discrepancy in pain behavior in the ipsilateral side of SNI, SNI + NS, and SNI + Res rats at other time points (Fig. 3b). Together, our data show that resveratrol partially reverses the development of mechanical allodynia induced by SNI.

### Resveratrol inhibits TREM2 and Iba + cell numbers in the SDH of SNI model

**Fig. 4:**
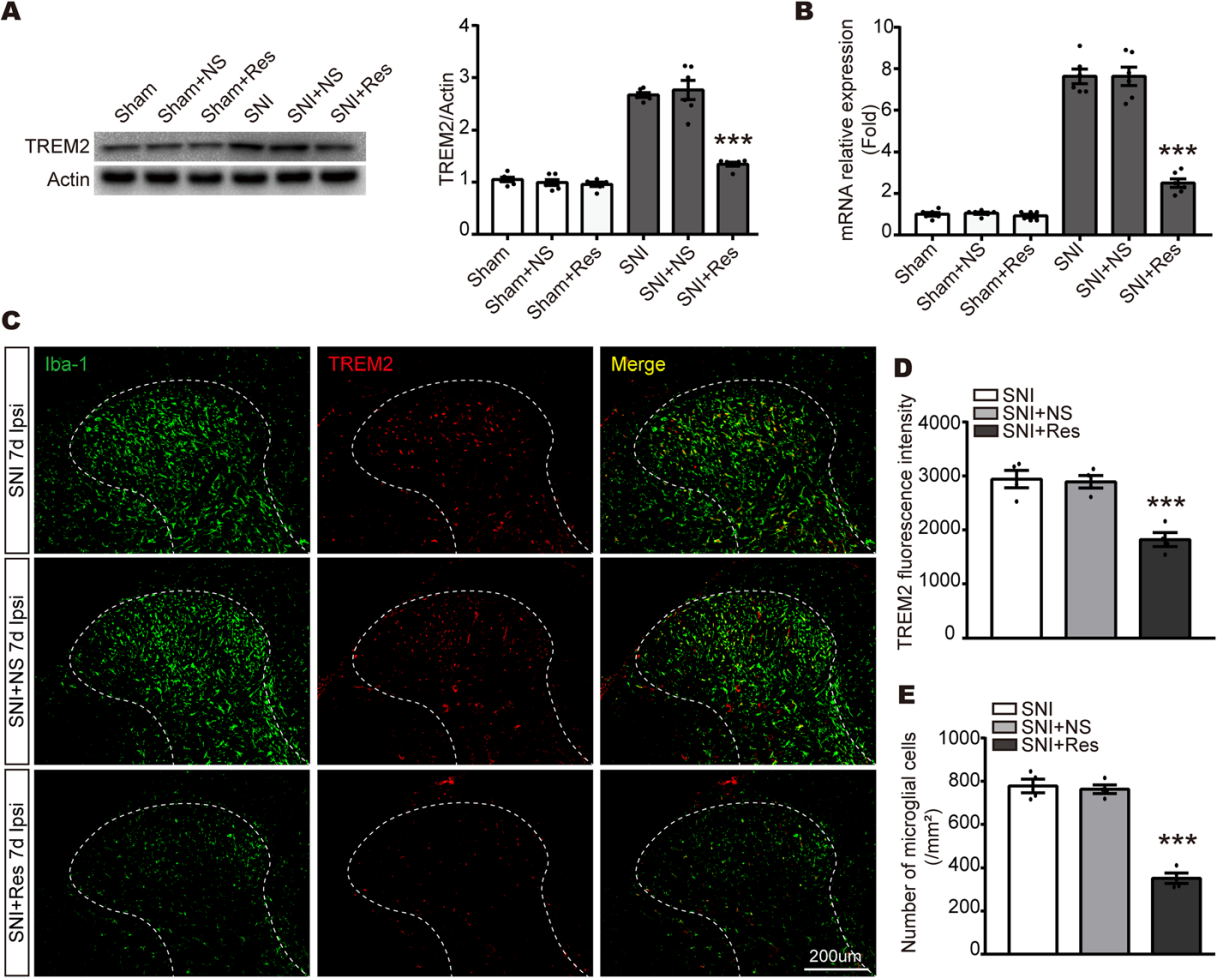
Resveratrol downregulates TREM2 and the number of microglial cells in NeuP states. **a** Representative blots and quantification of TREM2 in the ipsilateral SDH at 7 days in SNI rats after intrathecal administration of resveratrol or normal saline. **b** qPCR analysis of TREM2 expression in the ipsilateral SDH at 7 days in Sham and SNI rats after intrathecal administration of resveratrol or normal saline. **c** Representative confocal images of ipsilateral SDH showing double staining of TRME2 (red) and Iba-1 (green) at 7 days following SNI in SNI, SNI + NS, and SNI + Res rats. Original magnification, ×20; scale bar, 200 μm. **d**, **e** Summarized data for TREM2 intensity (**d**) and Iba1-positive microglial numbers (**e**) in the ipsilateral SDH from the three groups shown in (**c**) (*n* = 4 rats per group). Data were presented as the mean ± SEM. *n* = 6 per group. ***p < 0.001 vs. SNI + NS group

To investigate the association between resveratrol and TREM2 after peripheral nerve injury, TREM2 level was detected by qPCR and Western blot. As indicated in Fig. 4a, b, the mRNA and protein expression of TREM2 was drastically hampered in the ipsilateral SDH in SNI + Res group, compared to SNI + NS group at 7 days after SNI. However, the level of TREM2 was not significantly different in Sham rats after treatment with resveratrol or normal saline. Meanwhile, immunofluorescence staining further demonstrated that resveratrol reduced TREM2 expression in microglia in the ipsilateral SDH after SNI surgery (Fig. 4c, d). Moreover, the number of Iba1-positive microglia was decreased after administration of resveratrol (Fig. 4e). The similar results were observed in primary microglia (Fig. S2). Collectively, these results indicate that resveratrol–induced protection of neuropathic pain is likely associated with the reduction of TREM2 expressed in microglia.

### Resveratrol promotes autophagy and alleviates neuroinflammation in SNI rats

**Fig. 5:**
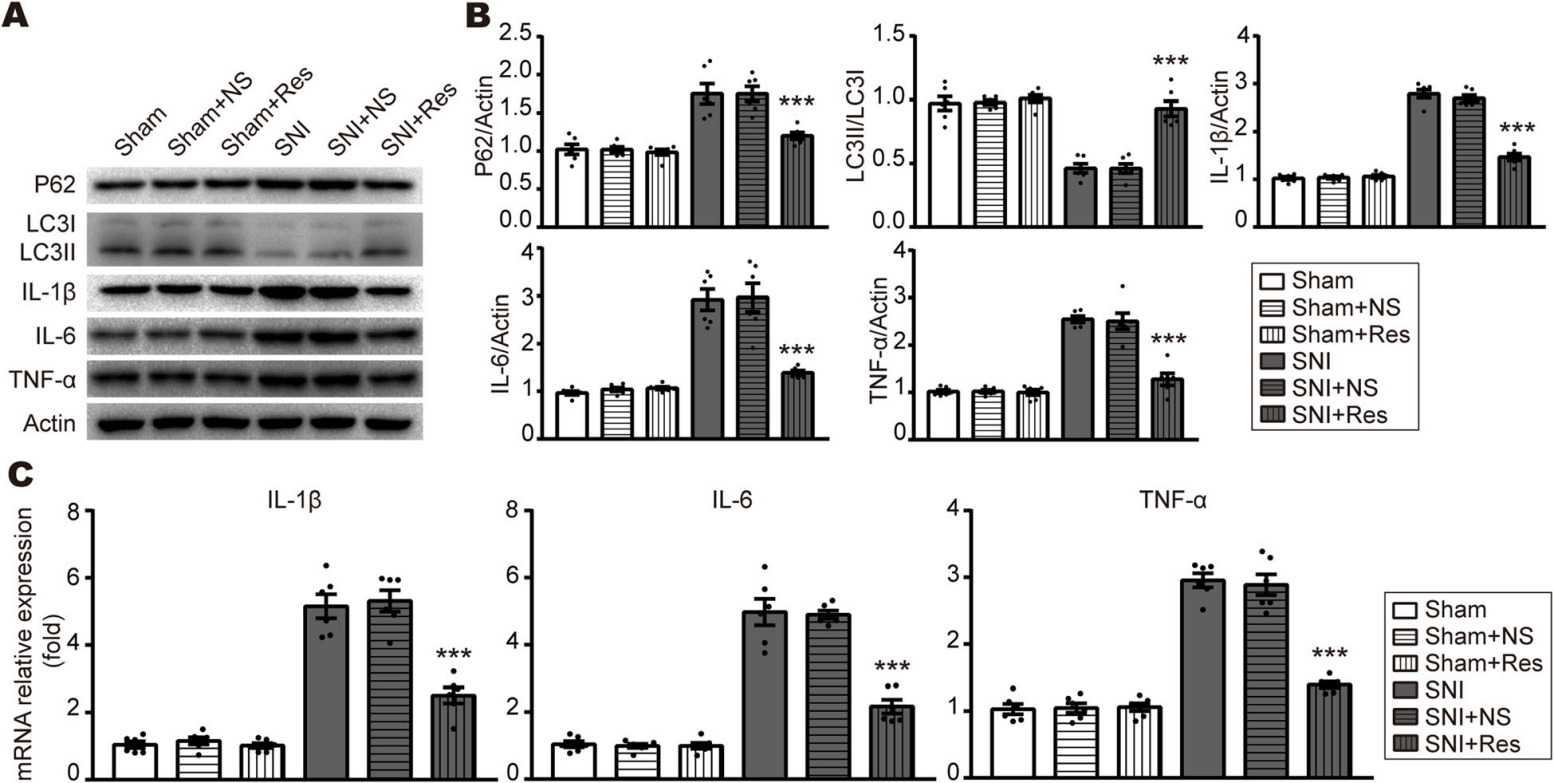
Resveratrol induces autophagy and inhibits the expression of inflammatory cytokines in SNI rats. **a**, **b** Representative Western blots and quantification of P62, LC3II/LC3I, IL-1β, IL-6, and TNF-α in the ipsilateral SDH at 7 days in Sham and SNI rats after intrathecal administration of resveratrol or normal saline. **c** The mRNA level of IL-1β, IL-6, and TNF-α in the ipsilateral SDH at 7 days in Sham and SNI rats after intrathecal administration of resveratrol or normal saline. Data were presented as mean ± SEM. *n* = 6 rats per group. ***p < 0.001 vs. SNI + NS

Neuroinflammation is an essential cause in the pathology of neuropathic pain. To characterize whether resveratrol regulated inflammatory response in the SDH of the SNI rat model, the expression of pro-inflammatory cytokines was measured by Western blot and qPCR. As shown in Fig. 5a-c, the protein and mRNA levels of IL-1β, IL-6, and TNF-α were markedly decreased in the SDH of SNI + Res group, compared with the SNI + NS group. However, the administration of resveratrol or normal saline did not change the levels of IL-1β, IL-6, and TNF-α in Sham rats. These results suggested that resveratrol alleviated mechanical allodynia by inhibiting the inflammatory response in the SDH after SNI surgery. Next, we determined whether resveratrol could impact autophagy in the SDH after SNI surgery. As shown in Fig. 5a, b, the expression of P62 was significantly restrained in the SDH, and the ratio of LC3II/LC3I was obviously increased in the SDH of SNI + Res group, compared to SNI + NS group, but the result in Sham rats was not obviously different in Sham rats treated compared with resveratrol or normal saline. These data imply that resveratrol alleviates mechanical allodynia by the promotion of autophagy and the restriction of neuroinflammation in SNI rats.

### Resveratrol reverses mechanical allodynia in SNI rats via TREM2-mediated autophagy

**Fig. 6:**
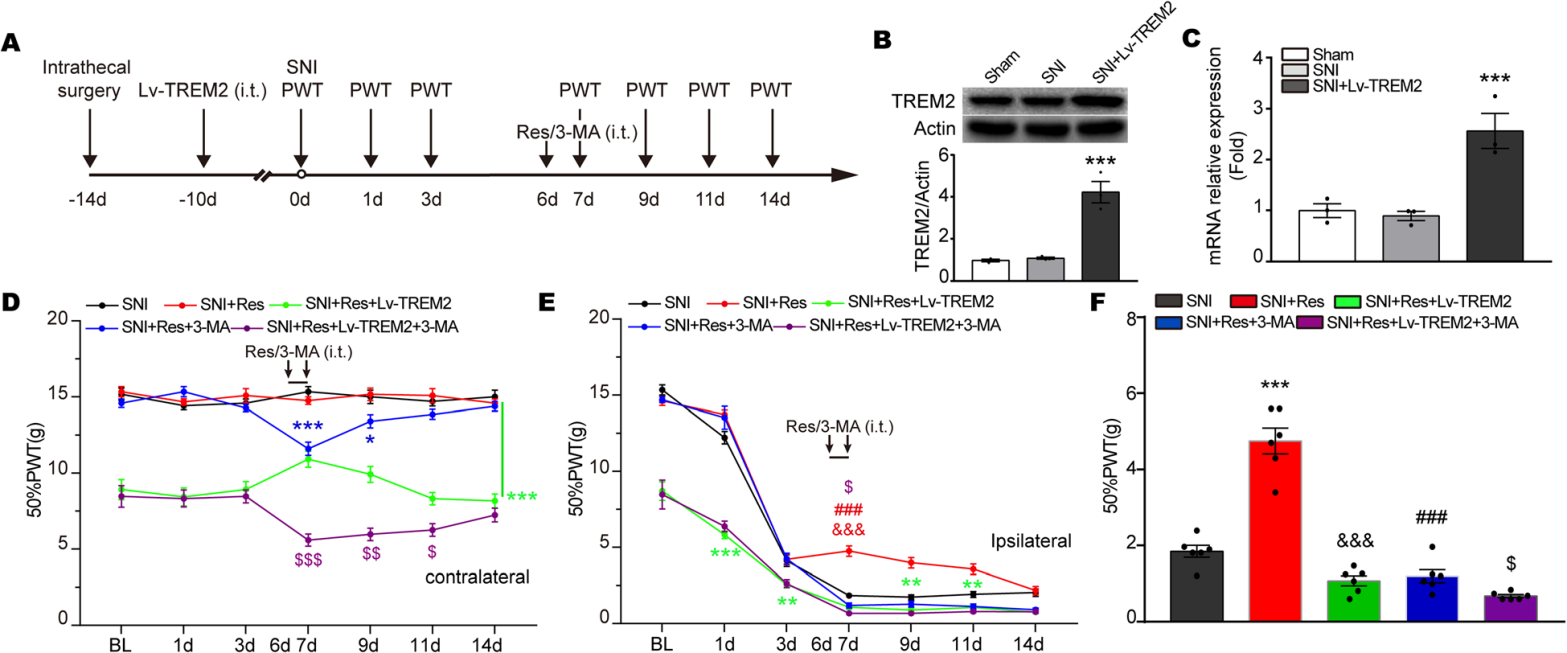
Resveratrol reverses mechanical allodynia in SNI rats via enhancing TREM2-mediated autophagy. **a** Schematic of experimental procedures. **b** Representative Western blots and quantification of TREM2 in the ipsilateral SDH at 7 days in the Sham, SNI, and SNI + Lv-TREM2 rats. **c** The mRNA level of TREM2 in the ipsilateral SDH at 7 days in the Sham, SNI, and SNI + Lv-TREM2 rats. **d**, **e** The mechanical allodynia of the contralateral (**d**) and ipsilateral side (**e**) was detected by von Frey test at indicated time points in SNI, SNI + Res, SNI + Res + Lv-TREM2, SNI + Res + 3-MA, and SNI + Res + Lv-TREM2 + 3-MA group. Data were presented as mean ± SEM. *n* = 5 rats per group. The same color represented the same group. ***p < 0.001 vs. SNI + Res group, &&&*p* < 0.001 vs. SNI + Res + Lv-TREM2 group, ###*p* < 0.001 vs. SNI + Res + 3-MA. $*p* < 0.05 vs. SNI + Res + Lv-TREM2. **f** The 50% PWT in the ipsilateral side on day 7 from the five groups shown in (**e**). Data were presented as mean ± SEM. *n* = 6 rats per group; ***p < 0.001, **p < 0.01 vs. SNI group, &&&*p* < 0.001 vs. SNI + Res group, ###*p* < 0.001 vs. SNI + Res. $*p* < 0.05 vs. SNI + Res + Lv-TREM2

To investigate whether resveratrol ameliorated mechanical allodynia in NeuP rats is mediated by the TREM2-induced autophagy axis, we activated TREM2 by i.t. injection of Lv-TREM2, or inactivated autophagy by i.t. injection of 3-methyladenine (3-MA, an inhibitor of autophagy) in resveratrol-treated SNI rats (Fig. 6a). The transfection effect of Lv-TREM2 was analyzed by Western blot and qPCR. As shown in Fig. 6b and c, the expression of TREM2 was significantly upregulated by treatment with Lv-TREM2. All groups were tested at 0, 1, 3, 7, 9, 11, and 14 days for mechanical allodynia using the von Frey test. A two-way repeated-measures ANOVA revealed significant differences across different days and groups. We found no obvious differences in the contralateral side at 7 days in SNI + Res rats versus SNI. The level of 50%PWT was decreased in the contralateral side of SNI + Res + 3-MA versus SNI + Res rats (Fig. 6d). Compared with the SNI + Res group, both contralateral and ipsilateral 50% PWT were decreased by Lv- TREM2 treatment at all tested days in SNI + Res + Lv-TREM2 or SNI + Res + Lv-TREM2 + 3-MA group. Resveratrol increased the 50% PWT on day 7 compared with day 3 in SNI + Res+Lv-TREM2 group (Fig. 6d). In the ipsilateral side, we found that the 50% PWT was significantly decreased on day 7 in SNI + Res + Lv-TREM2 group compared to SNI + Res, suggesting that Lv-TREM2 blocked the antinociceptive effect induced by resveratrol (Fig. 6e). The similarity is that an obvious decrease in the 50% PWT was shown at day 7 after SNI in SNI + Res + 3-MA rats versus SNI + Res rats, suggesting that 3-MA interrupted the antinociceptive effect triggered by resveratrol. In contrast to SNI + Res + Lv-TREM2 rats, the facilitation of TREM2-induced mechanical allodynia was further aggravated by the hindrance of autophagy with 3-MA administration in the ipsilateral side of SNI + Res + Lv-TREM2 + 3-MA rats (Fig. 6d, f). To sum up, these results suggest that resveratrol improves mechanical allodynia by regulating TREM2-mediated autophagy, and application of Lv-TREM2 and 3-MA blocked this effect.

### Resveratrol mitigates neuroinflammation by regulating TREM2-mediated autophagy

**Fig. 7:**
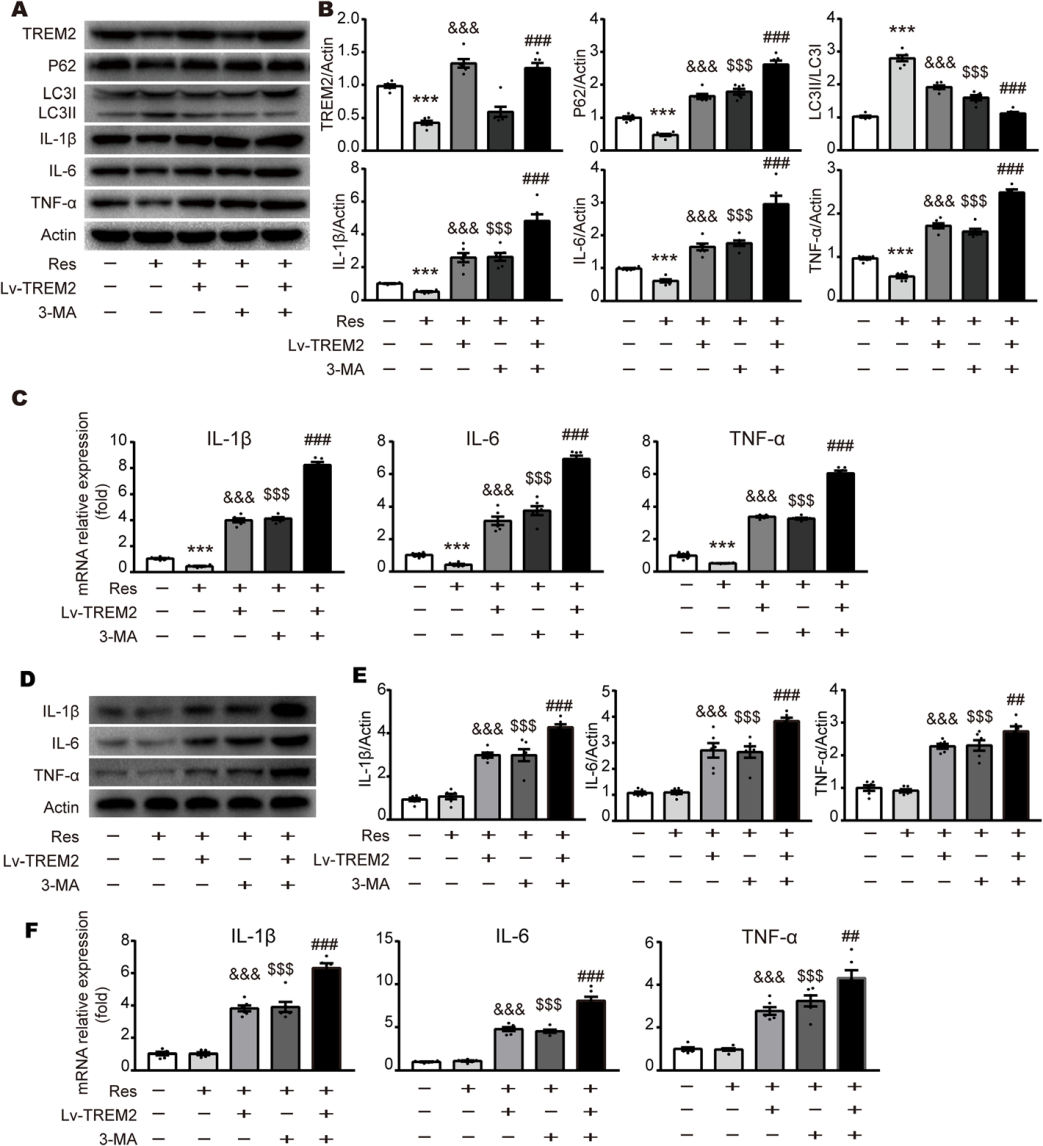
TREM2 or 3-MA reverses the anti-inflammation effects of resveratrol after SNI. **a**, **b** Representative blots and expression of TREM2, P62, LC3, IL-6, IL-1β, and TNF-α in the ipsilateral SDH at 7 days after SNI with i.t. resveratrol, Lv-TREM2, or 3-MA administration. **c** qPCR analysis of pro-inflammatory gene expression in the ipsilateral SDH at 7 days after SNI with i.t. resveratrol, Lv-TREM2, or 3-MA administration. **d**, **e** Representative blots and expression of IL-6, IL-1β, and TNF-α in the contralateral SDH at 7 days after SNI with i.t. resveratrol, Lv-TREM2, or 3-MA administration. **f** qPCR analysis of pro-inflammatory gene expression in the contralateral SDH at 7 days after SNI with i.t. resveratrol, Lv-TREM2, or 3-MA administration. Values were presented as the mean ± SEM. *n* = 6 per group. ***p < 0.001 vs. SNI group, &&&*p* < 0.001 vs. SNI + Res group, $$$*p* < 0.001 vs. SNI + Res group, ###*p* < 0.001 vs. SNI + Res + Lv-TREM2 group

To confirm the mechanism underlying the anti-inflammatory effects of resveratrol in SNI rats, we investigated the effects of TREM2 overexpression and/or 3-MA on the inflammatory response. Lv-TREM2 was intrathecally injected into SNI rats followed by evaluation with Western blot and qPCR. In the ipsilateral spinal dorsal horn at 7 d after SNI, the protein and mRNA levels of IL-1β, IL-6, and TNF-α were increased in SNI + Res + Lv-TREM2 rats compared with SNI + Res rats, suggesting that TREM2 contributed to inflammation. Namely, resveratrol inhibited inflammatory response by downregulating TREM2 (Fig. 7a-c). Levels of IL-1β, IL-6, and TNF-α were robustly increased in the contralateral side of SNI + Res + Lv-TREM2 versus SNI + Res group (Fig. 7d-f). As TREM2 has been shown to suppress autophagy, we asked whether resveratrol exerted a protective effect against neuroinflammation via the TREM2-autophagy axis. Western blot showed an increase in P62 and a decrease in the ratio of LC3II to CL3I in the SNI + Res + Lv-TREM2 group compared to the SNI + Res group. The upregulation of pro-inflammatory cytokines activated by Lv-TREM2 was increased to an even higher level after administration of 3-MA (Fig. 7a-c), indicating that TREM2-induced neuroinflammation after SNI in an autophagy-dependent manner. Thus, these data indicate that resveratrol improves neuroinflammation in a TREM2-autophagy dependent manner in SNI rats.

## Discussion

Here, we explored the effects of resveratrol on NeuP and its mechanisms of resveratrol-mediated neuroprotection in the spinal dorsal horn of spared nerve injury rats. Results from the current study show the association between resveratrol and TREM2/autophagy-mediated inflammation in NeuP. We employed a well-characterized model of neuropathic pain, a spared nerve injury rat model, with intrathecal manipulation of resveratrol or inflammation-associated molecules. Levels of TREM2, autophagy, and inflammation were significantly elevated in response to spared nerve injury. Interestingly, the levels of all three above and mechanical allodynia induced by SNI were significantly inhibited after the administration of resveratrol. Intrathecal injection of lentivirus containing TREM2 or 3-MA inhibited the TREM2-mediated autophagy and stimulated the secretion of TNF-α, IL-6, and IL-1β in SNI rats after resveratrol treatment. Taken together, our data revealed that resveratrol attenuated inflammatory response and mechanical allodynia in a TREM2/autophagy-dependent manner.

Microglia, innate immune cells in the CNS, are closely associated with the immune response in some neurodegenerative diseases, such as Parkinson’s diseases (PD), Alzheimer’s disease (AD), and seizure [29]. In recent years, there has been increasing evidence that microglia play a key role in the pathology of NeuP [30]. After peripheral nerve injury, injury information is transmitted to the spinal cord via afferent fibers and releases of colony-stimulating factor 1 (CSF1), chemokine and matrix metalloproteinase 9 (MMP-9) and also many other molecules. Microglia in the spinal dorsal horn detect and respond rapidly to nerve injury [31]. In our study, the expressions of IL-1β, IL-6, TNF-α, and the number of microglia were increased in the SDH of SNI rats. These results are in accordance with the prevailing view that microglia-mediated neuroinflammation is involved in NeuP [32]. Notably, the administration of resveratrol ameliorated mechanical allodynia and decreased the level of inflammatory cytokines in the SDH through inhibiting the microglia activation. Similar inhibitory effects of resveratrol on inflammation were observed in the nerve injury model lacking DAP12 or TREM2, and AD model [24, 33, 34]. These results suggested that microglia activation was inhibited by resveratrol in the pathology of NeuP.

Resveratrol, a flavonoid polyphenol compound extracted mostly from red wine or grapes, exerts a variety of beneficial effects, including anti-oxidant, anti-apoptosis, anti-cancer, and anti-inflammation [12]. Previous studies have shown that resveratrol has neuroprotective properties in NeuP, AD, and PD. It has been demonstrated that resveratrol attenuated inflammation by inhibiting the pro-inflammatory cytokine secretion in the obese model and cirrhotic model [35–37]. Administration of resveratrol elicits neuroprotective function via suppressing glial activation and decreasing the secretion of pro-inflammatory cytokines in PD mice [14, 38]. Moreover, recent studies have reported that resveratrol alleviated neuroinflammation in the spinal trigeminal nucleus of CCI rats via inhibiting microglia activation [38]. Similarly in the present study, we found that intrathecal administration of resveratrol exerted significant anti-inflammation effect by downregulating IL-1β, IL-6, and TNF-α, and inhibiting microglia activation in the SDH of SNI rats, and in turn ameliorated mechanical allodynia. Furthermore, resveratrol promoted autophagy by inhibiting the TLR4/NF-kB pathway, attenuating inflammation in TBI [39]. In our study, autophagy was activated after resveratrol treatment in SNI rats, which is implicated in anti-inflammation, reversed these effects by 3-MA. These data suggested that resveratrol inhibited microglia-mediated neuroinflammation by enhancing autophagy in the SDH of SNI rats. However, the mechanisms of suppressing microglia activation and enhancing autophagy induced by resveratrol remain unknown.

TREM2 is mainly expressed by microglia and implicated in various microglia function [5]. TREM2 has been investigated as a phagocytic receptor and immune receptor in microglia in the ischemia model and AD [40]. TREM2/DAP12 signal mediates microglia response by regulating inflammation in an AD model, and TREM2 deficiency exerts opposing effects on inflammation at early and late stages of AD, suggesting that TREM2 has different roles in different stages of the same diseases and different diseases [41]. Recent studies indicated that TREM2 elicits microglia-mediated neuroinflammation and exacerbates mechanical allodynia in spinal nerve injury mice [24]. Therefore, TREM2 is assumed to be an essential molecule that controls microglia activation status and the production of inflammatory cytokines from microglia. Based on the lines of evidence through multiple approaches including Western blot, qPCR, and immunofluorescence, we found that TREM2 expression was obviously increased in microglia in ipsilateral SDH of SNI rats. TREM2 overexpression reversed the neuroprotective effects of resveratrol, suggesting that TREM2 might be involved in microglia-induced neuroinflammation in SNI rats. Intrathecal administration of agonistic TREM2 antibody leads to injury-induced mechanical allodynia and neuroinflammation [24, 42]. These results are in line with our findings that overexpression of TREM2-induced neuroinflammation in SNI rats and reversed the neuroprotective effects of resveratrol treatment. In other words, these results indicate that resveratrol suppressed neuroinflammation and mechanical allodynia in SNI rats by downregulating TREM2 expression.

Autophagy is a lysosomal degradation pathway that eliminates damaged and redundant cellular components to maintain tissue homeostasis. The dysfunction of autophagy contributed to the development of diseases, including neurodegeneration, cancer, and NeuP [43]. Recently, accumulating studies revealed that the dysregulation of autophagy was observed in NeuP, which contributed to mechanical allodynia by triggering neuroinflammation [44]. Hydrogen-rich saline inhibited microglial activation, inhibiting inflammation in an autophagy-mediated manner [21]. More recent studies reported that TREM2-deficient microglia robustly induced autophagy via impairing mTOR signaling [5]. Here, we found that the inhibited autophagy in SNI rats was enhanced by resveratrol, whereas the enhanced autophagy induced by resveratrol could be abolished by TREM2 overexpression. Interruption of autophagy strengthens TREM2-associated function, indicating that TREM2 is involved in resveratrol-induced anti-inflammatory response via regulating autophagy in SNI rats.

There were some limitations to the current study. First, we concentrated on the anti-inflammation of resveratrol in SNI rats, but we did not investigate the other properties such as anti-oxidant and anti-apoptosis. Second, resveratrol-induced neuroprotection was not explored on neuron and astroglia in the present study. Third, autophagy was only inhibited by 3-MA, and the effect of autophagy inducer was not investigated in our study. Further studies are needed to explore the underlying mechanisms of the decreased TREM2 triggered by resveratrol.

## Conclusions

**Fig. 8:**
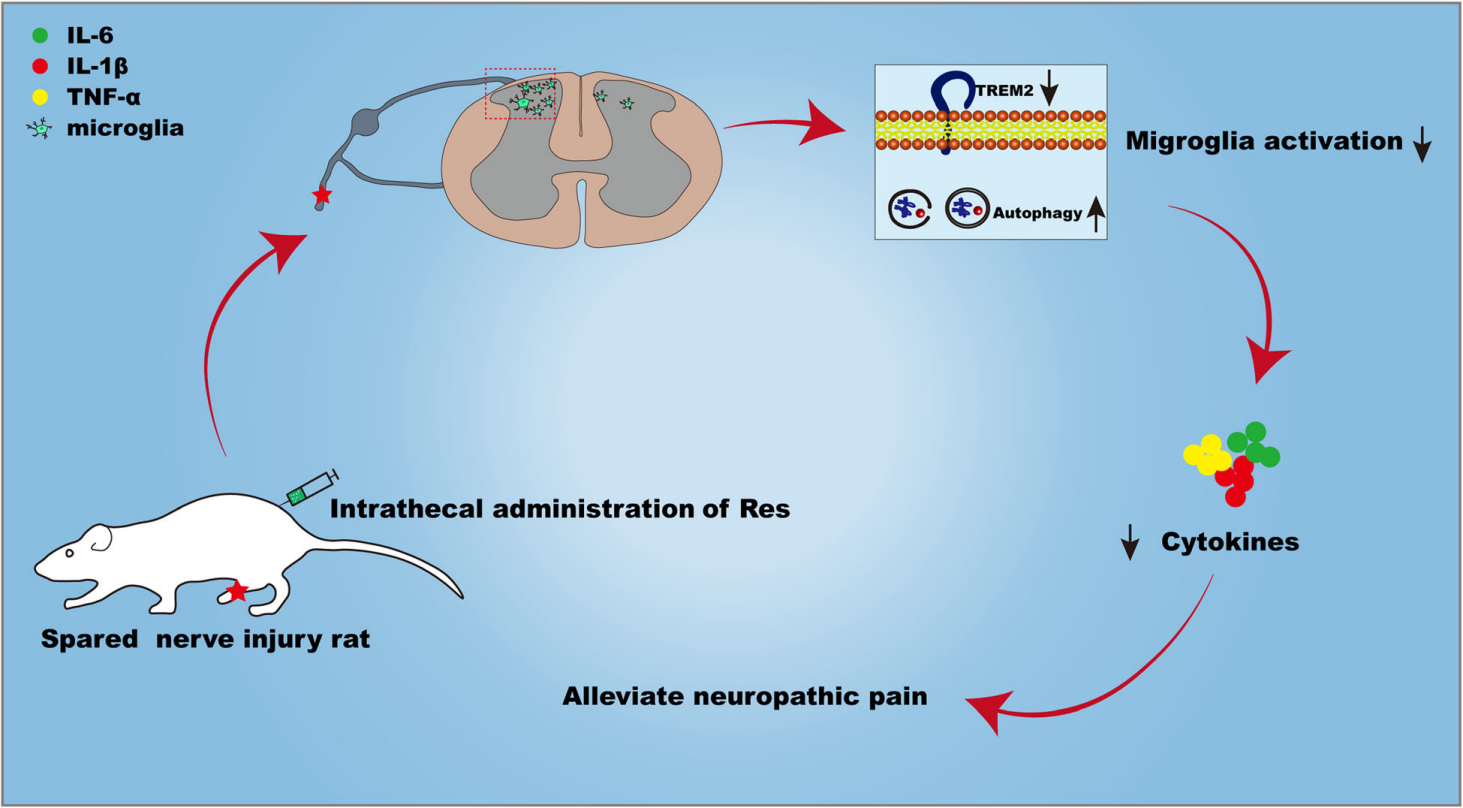
Schematic illustration of resveratrol-mediated protection of neuroinflammation. Resveratrol improves mechanical allodynia via inhibiting microglia-mediated neuroinflammation through the TRME2-autophagy axis after SNI surgery

Our experiment provides evidence that resveratrol has an effect on the TREM2-autophagy pathway, and resveratrol can alleviate mechanical allodynia by inhibiting neuroinflammation in the SDH (Fig. 8). In addition, our results demonstrated that TREM2-autophagy pathway participates in modulating the microglia function and regulating neuroinflammation to control the development of neuropathic pain in SNI rats. Although the explicit mechanism of resveratrol-mediated neuroprotection in neuropathic pain requires further studies, our findings also provide a possible therapeutic pathway to reduce inflammatory properties while improving mechanical allodynia in neuropathic pain.

## Supplementary information

**Additional file 1: Figure S1.** The expression of TREM2 is not colocalizated with neuron and astroglia. **A** Representative confocal images of double immunostaining showing colocalization of Neuron/GFAP (green) and TREM2 (red) signals in the ipsilateral SDH at 7d in SNI rats. Original magnification, 20x. Scale bar 100 μm; higher magnification, 25 μm.

**Additional file 2: Figure S2. Resveratrol induce the decreased of** TRME2 and the number of microglia in vitro. **A** Representative confocal images of double immunostaining showing colocalization of TREM2 (red) and TREM2 (green) signals. Original magnification, 40x. Scale bar 50 μm. **B** The quantification of TREM2 and number of microglia in Fig S2. Data were presented as the mean ± SEM. n = 5 per group. ***p<0.001 vs. Sham group, ###p<0.001 vs. LPS group.

## Data Availability

All data generated or analyzed during this study are included in this published article.

## Abbreviations

NeuP: Neuropathic pain
Res: Resveratrol
TREM2: Triggering receptor expressed on myeloid cells 2
SNI: Spared nerve injury
SDH: Spinal dorsal horn
PWT: Paw Withdrawal Threshold
3-MA: 3-Methyladenine
PVDF: Polyvinylidene fluoride
TBST: Tris-buffered-saline with tween
CNS: Central nervous system
Iba-1: Ionized calcium-binding adaptor molecule 1
NS: Normal saline
IL-1β: Interleukin-1 beta
TNF-α: Tumor necrosis factor alpha; IL-6: interleukin-6
