# Supplementary figures and images for: Resveratrol mediates mechanical allodynia through modulating inflammatory response via the TREM2-autophagy axis in SNI rat model
Source: J Neuroinflammation. 2020 Oct 21;17:311. doi: 10.1186/s12974-020-01991-2 (PMC7576710; doi:10.1186/s12974-020-01991-2)

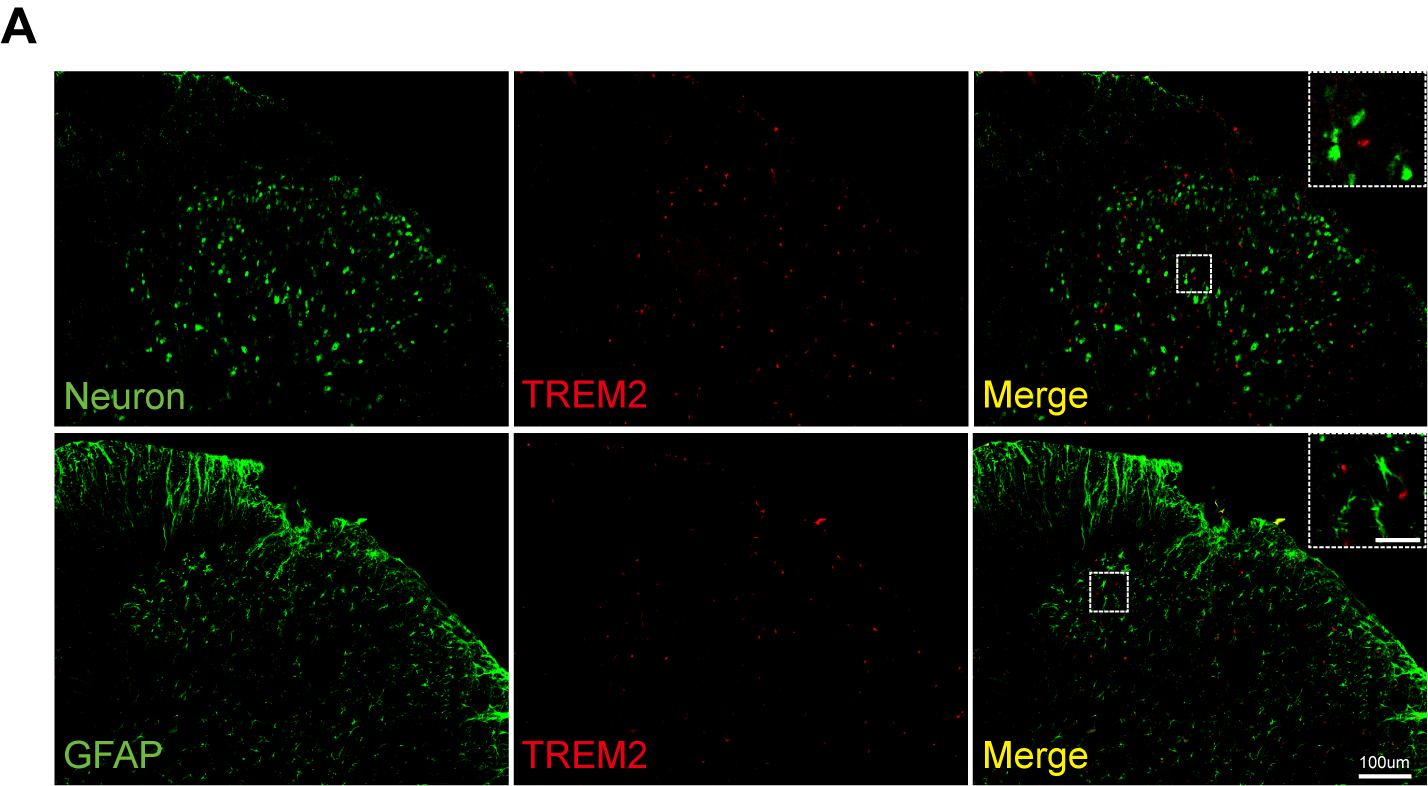

Supplement: Supplementary file 1 — Additional file 1: Figure S1. The expression of TREM2 is not colocalizated with neuron and astroglia. A Representative confocal images of double immunostaining showing colocalization of Neuron/GFAP (green) and TREM2 (red) signals in the ipsilateral SDH at 7d in SNI rats. Original magnification, 20x. Scale bar 100 μm; higher magnification, 25 μm. [file 12974_2020_1991_MOESM1_ESM.tif]

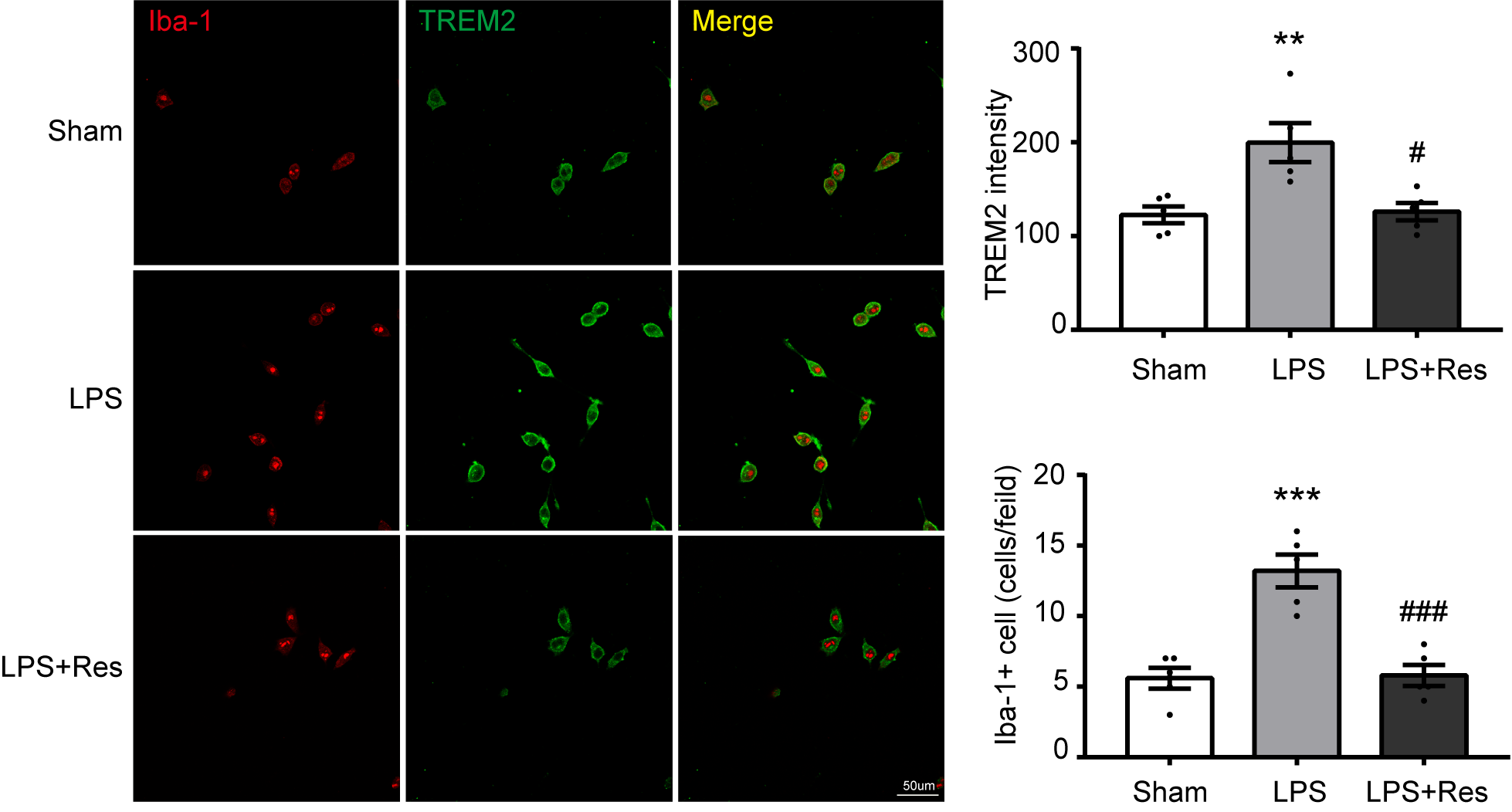

Supplement: Supplementary file 2 — Additional file 2: Figure S2. Resveratrol induce the decreased of TRME2 and the number of microglia in vitro. A Representative confocal images of double immunostaining showing colocalization of TREM2 (red) and TREM2 (green) signals. Original magnification, 40x. Scale bar 50 μm. B The quantification of TREM2 and number of microglia in Fig S2. Data were presented as the mean ± SEM. n = 5 per group. ***p<0.001 vs. Sham group, ###p<0.001 vs. LPS group. [file 12974_2020_1991_MOESM2_ESM.tif]
